# Ventricular Septal Defect in an Octogenarian: A Case Report of VSD Surgical Repair Concomitant with Coronary Artery Bypass and Valvular Surgery

**DOI:** 10.1155/2012/429569

**Published:** 2012-06-28

**Authors:** Eiki Tayama, Satoshi Fujita, Tomohiro Ueda, Ken-ich Imasaka, Naofumi Enomoto, Hirofumi Onitsuka, Yukihiro Tomita

**Affiliations:** Department of Cardiovascular Surgery, Clinical Research Center, Kyushu Medical Center, National Hospital Organization of Japan, Fukuoka 810-8563, Japan

## Abstract

Finding an untreated or asymptomatic large ventricular septal defect (VSD) in an elderly patient is uncommon. The present case was an 81-year-old man who suffered from acute myocardial infarction due to three-vessel coronary disease, mitral and tricuspid valve insufficiency, and high-flow perimembranous VSD (Qp/Qs 2.3). Although the patient was elderly and the VSD had been asymptomatic for a long time, we considered that high-flow VSD and valve diseases should be repaired simultaneously with coronary disease. Then, he underwent elective surgery, namely, VSD patch repair concomitant with coronary artery bypass grafting, and mitral and tricuspid annuloplasty. His postoperative course was uneventful. We conclude that, even for an octogenarian, surgical repair of VSD is recommendable, if surgical indications are appropriate.

## 1. Introduction

 Ventricular septal defect (VSD) is the most frequent congenital heart disease in adults, with equal distribution in both sexes, and it has an incidence of about 20% in children with heart defects [1, 2]. It is estimated that 25–40% of VSDs will close by the age of 2 years, and they are unlikely to persist after the age of 10 years [3]. Thus, in adults with congenital heart disease, VSDs represent only about 10% of the cases [1, 2]. Most large VSD are surgically corrected early in life because of the various cardiac events they induce. So, the finding of an asymptomatic large VSD in an elderly patient is extremely uncommon.

Here we report a surgical treatment experience in an 81-year-old patient who suffered from heart failure due to acute myocardial infarction and went on to be diagnosed with valvular disease and VSD. He underwent VSD patch repair concomitant with coronary artery bypass graft (CABG) ×3, mitral valve annuloplasty (MAP), and tricuspid valve annuloplasty (TAP).

## 2. Case Report

 An 81-year-old patient was referred to our hospital due to suffering from shortness of breath and orthopnea lasting several days. The patient reported having systolic murmur since childhood that had been originally diagnosed as a valvular disease and later as VSD. He had been working as a medical doctor without any cardiac symptoms until this event. He had had medical treatment for renal hypertension and mild bilateral atherosclerotic obstruction since 4 years prior. However, surgical repair of the VSD had not been recommended because of its asymptomatic character.

 When he was referred to our hospital, his condition was as follows.

**Heart and Respiratory Sound**

High-frequency Levine IV/VI holosystolic murmur was mainly audible in the left parasternal to apex lesion which was accompanied by a palpable thrill. Moist rales were heard in the bilateral lower lung field.

**Chest X-Ray**

**Figure 1:**
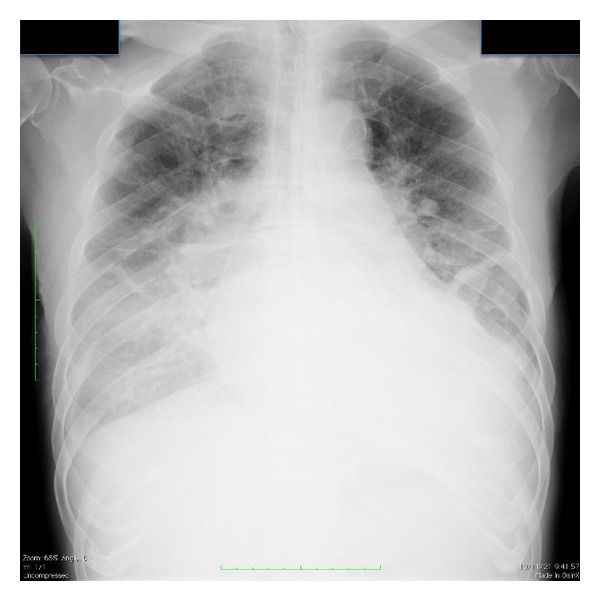
Chest X-ray at admission: significant pulmonary congestion and reduced bilateral lung permeability due to pleural effusion were seen (cardiothoracic rate of 62%).

Significant pulmonary congestion and reduced bilateral lung permeability due to pleural effusion were seen (cardiothoracic rate of 65%) (Figure 1).

**Electrocardiogram**

Normal sinus rhythm, 55 bpm. QS pattern in V1, V2; ST depression in V5, V6.

**Echocardiography and Continuous-Wave Doppler**

**Figure 2:**
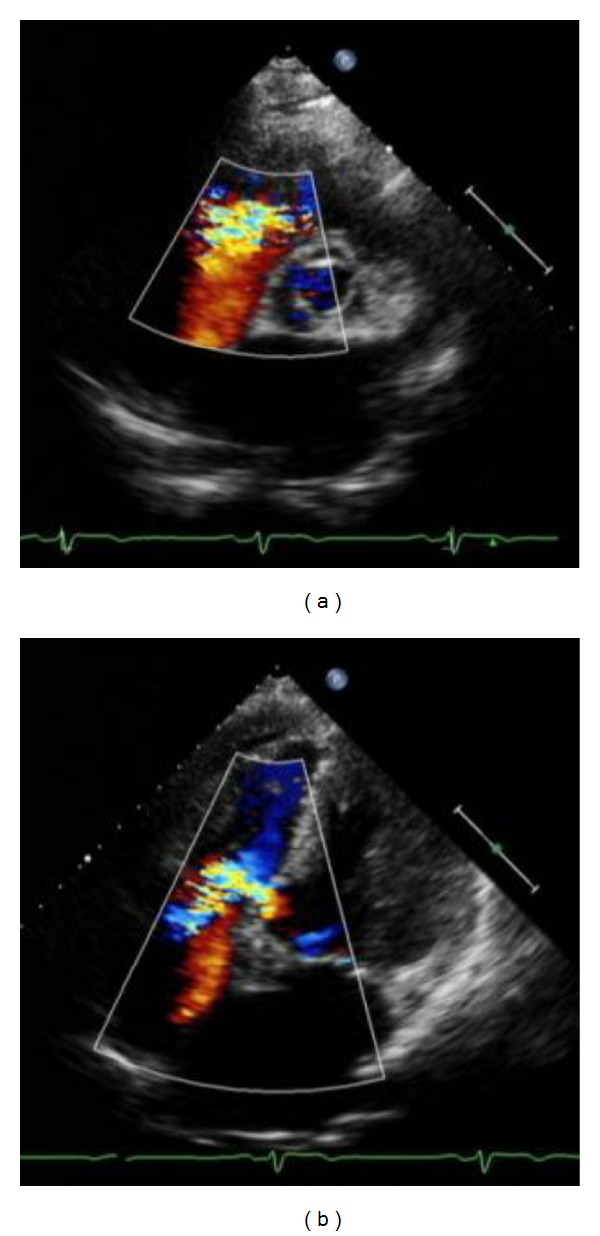
Echocardiography: short axis (a) and apical 4-chamber (b) view. Perimembranous ventricular septal defect *L* → *R* flow and moderate tricuspid regurgitation flow were seen.

Left to right shunt signal and perimembranous inlet type VSD were seen (high-velocity; maximum 3.9 m/s; pressure gradient of 60 mmHg) (Figure 2). The 5 mm of VSD's orifice was identified in the thickened membranous septal aneurysm. Severe tricuspid valve regurgitation (TR) was also detected (pressure gradient of RA-RV of 71 mmHg). Moderate mitral valve regurgitation (MR; area of 5 cm^2^ existed in a central portion. The left ventricle was slightly dilated and its motion showed severe hypokinesis in the anteroseptal to lateral area (LVDd/Ds 55/39 mm, LVEF 55%).

**Blood Examination**

Normal to slight increase of myocardium deviation enzyme (CK 80 IU/l, CK-MB 26 IU/l, LDH 342 mg/dL, AST 26 IU/l) and increased BNP 775 pg/mL. Both Troponin-T and heart type fatty acid-binding protein were positive. Renal function was slightly decreased (BUN 23 mg/dL, Cr 1.4 mg/dL).

After medical treatment for congestive heart failure, cardiac catheterization and coronary angiography were performed.

**Cardiac Catheterization**

Moderate pulmonary hypertension (46/17 mmHg, mean 28 mmHg) and significant increase of the pulmonary to systemic cardiac output (Qp/Qs of 2.3, Cardiac Output 4.66 L/min, Cardiac Index 2.84 L/min/m^2^).

**Coronary Angiography**

There were multiple stenotic lesions, including right coronary artery (RCA) no. 1 99%, no. 2 99%, no. 3 90%, no. 4AV 75%; left coronary artery (LCA) no. 6 99% (culprit lesion), no. 8 90%, no. 9 90%, no. 12 50%, no. 13 50%.

So, the final preoperative diagnoses were as follows: (1) acute myocardial infarction (anteroseptal) due to three-vessel disease; (2) perimembranous VSD; (3) moderate MR; (4) severe TR; (5) pulmonary hypertension.

 Although the patient was elderly and the VSD had been asymptomatic for a long time, we considered that high-flow VSD and valve diseases should be repaired simultaneously with coronary disease.

Thus, after medical control for congestive heart failure, we performed, as an elective operation, coronary artery bypass grafting (CABG), mitral annuloplasty (MAP), tricuspid annuloplasty (TAP), and VSD patch repair.

## 3. Operative Procedure

**Figure 3:**
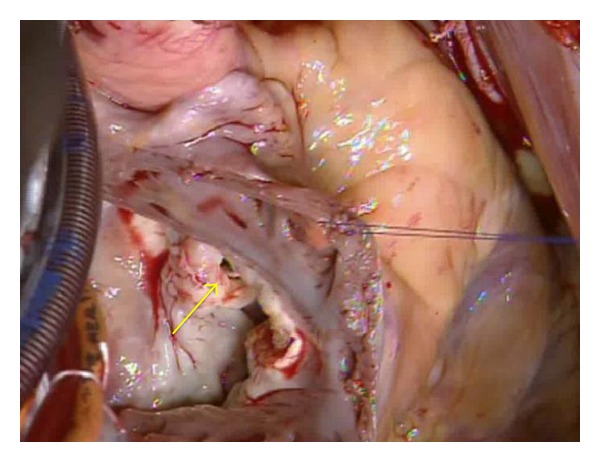
Perimembranous inlet-type VSD and membranous aneurysm (↑). The right-upper edge of the membranous aneurysm was tightly adhered and formed a mass with part of the tricuspid septal leaflet. The diameter of the VSD orifice was 8 mm while the original VSD size was speculated to be at least 15 × 20 mm.

 The left internal thoracic artery (LITA) and saphenous vein graft (SVG) were harvested. A cardiopulmonary bypass was established with superior and inferior vena cava drainage, and ascending aorta perfusion, as usual. There was a mottled-like scar in the anterior-posterior left ventricle due to myocardial infarction. Following ascending aorta cross-clamp and antegrade cardioplegia administration, we performed distal anastomosis for a three-coronary-artery bypass; SVG to RCA no. 4 PD, SVG to no. 14PL, and LITA to LAD no. 8. Then, a right atrial incision was made and retrograde cardioplegia was initiated. Both anterior and septal leaflets of the tricupid valve were partially thickened and their surface had become rough, as a result of high-flow VSD and TR jets. The right-upper edge of the membranous aneurysm was tightly adhered and formed a mass with a part of tricuspid septal leaflet. The VSD was a perimembranous inlet-type. Its entire circumference had become a solid membranous aneurysm. The diameter of the VSD orifice was 8 mm and the original VSD size was speculated to be at least 15 × 20 mm. (Figure 3).

 The VSD was closed using a Goretex sheet (15 × 15 mm round shape, thickness 0.4 mm) with mattress sutures. The mitral valve was examined through the septal approach. There was no leaflet prolapse or chorda rupture but annular dilatation. Then, MAP was performed with a Carpentier Edwards Physio Ring II of 28 mm (Edwards Lifescience, Irvine, CA). Following declamping of the ascending aorta, TAP with an Edwards MC^3^ ring of 30 mm (Edwards Lifescience, Irvine, California) and proximal SVG anastomosis were performed. The patient was weaned from cardiopulmonary bypass without difficulty.

 The operation time was 8 hour 14 min; the aortic cross clamp time, 175 min; the cardiopulmonary bypass time, 257 min; his lowest rectal temperature, 32.1°C.

The patient's postoperative course was uneventful. No residual VSD or MR was recognized by echocardiogram. Postoperative CT demonstrated that all coronary artery grafts were patent. He was discharged on postoperative day 16 and has been well for more than 1 year now.

## 4. Discussion

Currently, cardiac surgery for patients over 80 years in age is not uncommon, including CABG and valvular operation. However, case reports are rare in the surgical management of congenital heart disease, such as VSD, in octogenarians. The present case is the first surgically treated case for VSD concomitant with coronary and valvular diseases in an octogenarian.

 The natural history of VSDs is largely dependent on the size and location of the defect and the downstream pulmonary vascular resistance [4]. Thus, patients with large VSDs in adult life present with congestive heart failure or pulmonary hypertension and right heart failure; thus, closure would be undergone in early life, while adults with small defects are usually asymptomatic. In addition, it is not easy to obtain accurate data of relatively asymptomatic congenital heart disease after patients become adults. One of the major reasons are the following doctor changes from a pediatric cardiologist to an adult cardiologist. So, evidence in the medical literature concerning elderly patients with VSD is lacking, particularly for those over 80 years old (5, 6, 7); one of the three was a finding on autopsy [5].

In this particular case, congestive heart failure due to acute coronary disease triggered an opportunity to clarify MR, TR, and a large VSD. Precisely speaking, the VSD had been pointed out in the patient's younger days; however, it had been left untreated because it was asymptomatic. Since he was over 80 years old, we had to carefully consider the surgical indication and strategy for VSD.

 Generally, the surgical indications for VSD in adults are large defects with pulmonary to systemic output Qp/Qs > 1.5/1, pulmonary hypertension >50 mmHg, progressive dilatation of the left atrial or the LV, reduced LV function, aortic regurgitation with perimembranous VSD, and a history of endocarditis, especially recurrent [1].

 However, for elderly VSD patients, the surgical indication is less clear. A conservative follow-up is often recommended unless a significant heart failure is revealed, with prophylaxis against infective endocarditis [6]. But, one must recognize that with a progressive increase in systemic vascular resistance with age, the degree of shunt can increase, making VSDs become more hemodynamically significant later in life [4]. So, some surgeons recommend VSD closure as long as pulmonary vascular obstructive disease is not prohibitive [4].

 In this case, off-pump CABG, which means leaving VSD and/or valvular disease, was another possible surgical option to avoid too extensive treatment. However, despite its asymptomatic history, high-flow VSD, in addition to MR and TR, would likely lead to perioperative and future heart failure. Fortunately, his physical condition was considered able to withstand a complex operation; thus, we decided to perform CABG, MAP, TAP, and VSD repair simultaneously. His postoperative course was uneventful.

By the way, the number of adult CHD cases having coronary artery disease have increased [7, 8], though cyanosis may exert a protective effect against coronary atherosclerosis [9–11]. The enormous growth in the number of adult CHD cases are a consequence of advances in surgical technique, intensive care, and noninvasive diagnosis. Giannakoulas et al. reported that 250 adult CHD cases (mean age of 51 ± 15 yo) underwent selective coronary angiography; 9.2% of adult patients demonstrated significant coronary artery disease [7]. Cui et al. reported that 3.4% of 852 adults over the age of 40 undergoing correction of a congenital anomaly required a concurrent CABG [8]. Coronary artery disease increases with advancing age and in combination with predisposing factors, such as hypertension, hyperlipidemia, and diabetes mellitus. They recommend coronary angiography examination for patients over the age of 50, as well as for patients younger than 50 when CAD predisposing factors are present [9]. In the near future, adult congenital heart disease patients who need coronary revascularization will increase in number, regardless of whether their congenital deficits have already been repaired or not.

## 5. Conclusion

 In an 81-year-old patient having coronary and valvular disease and VSD, we performed surgery addressing these issues simultaneously. Even in such an elderly case, VSD repair is recommendable if its surgical indication is appropriate.
